# AMDCnet: attention-gate-based multi-scale decomposition and collaboration network for long-term time series forecasting

**DOI:** 10.3389/frai.2025.1607232

**Published:** 2025-05-30

**Authors:** Shikang Hou, Song Sun, Tao Yin, Zhibin Zhang, Meng Yan

**Affiliations:** ^1^School of Big Data and Software Engineering, Chongqing University, Chongqing, China; ^2^School of Computer and Information Science, Chongqing Normal University, Chongqing, China

**Keywords:** long-term time series, forecasting, multi-scale decomposition, feature fusion, attention-gate

## Abstract

**Introduction:**

Time series analysis plays a critical role in various applications, including sensor data monitoring, weather forecasting, economic predictions, and network traffic management. While traditional methods primarily focus on modeling time series data at a single temporal scale and achieve notable results, they often overlook dependencies across multiple scales. Furthermore, the intricate structure of multi-scale time series complicates the effective extraction of features at different temporal resolutions.

**Method:**

To address these limitations, we propose AMDCnet, a multi-scale-based time series decomposition and collaboration network designed to enhance the model's capacity for decomposing and integrating data across varying time scales. Specifically, AMDCnet transforms the original time series into multiple temporal resolutions and conducts multi-scale feature decomposition while preserving the overall temporal dynamics. By extracting features from downsampled sequences and integrating multi-resolution features through attention-gated co-training mechanisms, AMDCnet enables efficient modeling of complex time series data.

**Results:**

AMDCnet achieving 44 best results and 10 second-best results out of 64 cases. Experimental results on 8 benchmark datasets demonstrate that AMDCnet achieves state-of-the-art performance in time series forecasting.

**Discussion:**

Our research provides a robust baseline for the application of artificial intelligence in multivariate time series forecasting. This work leverages multi-scale time series decomposition and gated units for feature fusion, effectively capturing dependencies across different temporal scales. Future studies may further optimize the scale decomposition and fusion modules. Such efforts could enhance the representation of multi-scale information and help address key challenges in multivariate time series prediction.

## 1 Introduction

In recent years, accurate long-term time series forecasting has become increasingly important across various fields, including finance (D'Urso et al., 2021), healthcare (Bahadori and Lipton, 2019), energy management (Zhou et al., 2021), and weather prediction (Wu et al., 2021). Time series data inherently exhibit variability over time, characterized by continuity, trends, and periodicity; this complexity and uncertainty present significant challenges. Consequently, deep learning models, which are adept at capturing nonlinear relationships and intricate temporal features, have gained widespread application in time series forecasting tasks.

Existing deep learning frameworks for long-term time series forecasting can be categorized into three groups: (i) Recurrent Neural Network (RNN)-based models (Connor et al., 1994); (ii) Transformer-based models (Vaswani, 2017); and (iii) Temporal Convolutional Networks (TCNs)-based models (Bai et al., 2018). RNN-based models have evolved various variants, such as Long Short-Term Memory (LSTM) (Hochreiter, 1997), Gate Recurrent Unit (GRU) (Cho, 2014). LSTM solves the problem of gradient vanishing but still faces the problem of long training time and difficulty in capturing the production time dependence, GRU is faster in training compared to LSTM but still has limitations in dealing with long sequences; Transformer-based models rely on the self-attention mechanism to model the long-distance relationship effectively, but it requires a large amount of computational resources when dealing with long sequences; TCNs-based model expands the receptive field by stacking convolutional layers, which can effectively extract local and global information, but sometimes relies too much on local features and faces the problem of large memory usage when dealing with long sequences. Existing studies have applied multi-view learning (Yu et al., 2025) and broad learning (Lin et al., 2025; Zhong et al., 2024) to anomaly detection or time series forecasting. ILMNN (Yang et al., 2024) mitigates intra-class imbalance by reducing intra-class sample distances while increasing inter-class sample distances, combined with weight allocation. GEIB (Chen et al., 2025) enhances system robustness by employing an adaptive broad learning system to capture variability among imbalanced samples.

These models hold great potential for time-series modeling. However, they often fail to account for feature learning across different time scales, which results in suboptimal capturing of complex dataset characteristics. To address this issue, recent approaches have employed multi-scale feature learning frameworks to capture intricate data patterns. For example, TimesNet (Wu et al., 2022) addresses the limitations of one-dimensional data representation by transforming one-dimensional time data into two-dimensional tensors, thereby capturing variations within and across periods. MSGNet (Cai et al., 2024) aims to capture the correlations between multi-dimensional time data sequences by utilizing frequency domain analysis and adaptive graph convolutions across multiple scales to capture specific and comprehensive inter-sequence dependencies, while also integrating self-attention mechanisms to model intra-sequence dependencies. Although these multi-scale methods have demonstrated commendable performance in time-series forecasting and validated the effectiveness of multi-dimensional data from different modeling perspectives, they overlook further decomposition of the sequence during the multi-scale partitioning phase. Multi-scale learning is performed only at the sequence level, which, while effectively learning periodic information, may neglect trend-related information in the data. For instance, when sequences exhibit trend-like changes over an extended time span, the limitations of the multi-scale range might hinder the attention mechanism from capturing meaningful information.

All methods encounter two key challenges: (i) multivariate time series often exhibit complex characteristics such as randomness and periodicity, which cannot always be simplified into seasonal and trend components; (ii) simply combining forecasting results fails to highlight the primary features of the time series. To address these issues, we propose a multi-scale decomposition and collaboration network based on attention gates. AMDCnet is not limited to capturing only seasonal and trend patterns. After multi-scale decomposition, it employs odd-even sequence sampling to comprehensively cover sequence information. It then performs stacked learning on odd-even sequence information across different scales to capture a broader range of data characteristics. Subsequently, we employ an attention-gated collaborative module to learn time-series features at various resolutions and introduce a feature pyramid for more comprehensive feature fusion. Our method aims to model temporal dependencies across different scales in multi-dimensional time-series data through detailed decomposition and collaboration processes. The main contributions of this paper are as follows:

- We propose AMDCnet, a novel multiscale-based time series forecasting model that effectively creates an accurate temporal representation for multidimensional time series data by capturing sequence patterns without temporal resolution.
- We propose a multi-scale time series feature decomposition and collaboration module to explore the dependence of data features at different temporal resolutions and an attention-gate-based unit to promote the collaboration of different time scale representations, effectively modeling multi-scale temporal dependence.
- We validate AMDCnet using eight real-world datasets, and the experimental results show that it achieves better performance than recent state-of-the-art methods.

## 2 Related works

### 2.1 Time series forecasting

Research on time series forecasting tasks has been conducted for decades with a focus on time-varying modeling. Traditional methods such as Autoregressive Integrated Moving Average Model (ARIMA) (Box and Jenkins, 1968), which combines the three components of autoregression (AR) (Hannan and Quinn, 1979), differencing (I) (Dickey and Pantula, 1987) and moving average (MA) (Said and Dickey, 1984), can deal with non-stationary time series and capture trends, cycles and stochastic fluctuations in the time series, but real-world time series variations are very complex, and ARIMA's assumption that the time series is linear does not deal well with nonlinear and complex patterns.

In recent years, deep learning methods have effectively captured nonlinear and complex dependencies in time series data. RNN-based approaches, such as LSTM networks, adeptly handle long-term dependencies, while GRUs demonstrate superior efficiency. The NGCU (Wang et al., 2022) introduces a novel gating unit that enhances computational complexity and model sensitivity compared to LSTM and GRU, addressing the gradient vanishing and exploding issues common in traditional RNNs. Transformer-based methods excel at capturing dependencies over medium to long distances by utilizing self-attention mechanisms, significantly enhancing modeling capabilities for long sequences. FEDformer (Zhou et al., 2022) integrates seasonal-trend decomposition methods to improve the Transformer's performance in long-term forecasting by capturing the global profile of time series data. TimesNet (Wu et al., 2022) overcomes limitations of one-dimensional representation by transforming one-dimensional time data into a two-dimensional tensor, extracting intra-period and inter-week variations. The Temporal Convolutional Network (TCN)-based method does not rely on time-dependent sequential processing, instead capturing time series patterns through convolutional operations. M-TCN (Wan et al., 2019) constructs a sequence-to-sequence framework for non-periodic datasets, effectively improving prediction accuracy for multivariate time series. PSTA-TCN (Fan et al., 2023) employs a stacked TCN backbone network combined with a parallel spatio-temporal attention mechanism to extract features, significantly reducing computation time while enhancing prediction accuracy.

It is worth noting that while all these methods effectively enhance prediction performance, they do not take into account the potential benefits of combining the decomposition and collaboration of multi-scale features to further improve model efficacy.

### 2.2 Multi-scale feature learning

Multiscale feature learning is a crucial method in time series analysis, aiming to extract multiple features from a time series across different time scales or frequency ranges. Numerous recent approaches in time series analysis have integrated multiscale feature learning as a key component of their models. MANF (Feng et al., 2023) combines relative positional information with multiscale attention, recognizing that small-scale attention is more sensitive to local contexts while capturing higher-order global information through cascading attention mechanisms applied at hierarchical time scales, such as intra-day, intra-week, and Intra-month. MICN (Wang et al., 2023) introduces a multiscale hybrid decomposition module to separate seasonal and trend-periodic patterns in the series, but it only uses a simple mean operation to integrate these patterns, without considering an appropriate method for weight assignment. Preformer (Du et al., 2023) proposes a novel multiscale segmentation mechanism that encodes the series based on segmental correlation attention, aggregating dependencies across different time scales within a multiscale structure. These models have not explored the temporal features under finer variations of decomposition factor sizes, focusing only on feature learning at the sequence level. Moreover, these multi-scale techniques face challenges during the final scale fusion process, as they simply concatenate features without emphasizing the tendencies of the time series toward specific characteristics. How to reasonably allocate fusion weights across different scales has a critical impact on the accuracy of multivariate time series forecasting.

## 3 Methodology

### 3.1 Problem definition and formulation

In the context of multivariate time series forecasting, we consider a system containing *N* variables. Where the historical data is provided through a backward-looking window *X*_*t* − *L*:*t*_ of length *L*, this matrix includes the observed values of each variable from time point *t* − *L* to *t* − 1. The task of time series forecasting is to estimate the values of these variables at the next *T* time steps based on this historical data. The output is the prediction matrix ${\hat{X}}_{t\text{ to }t+T-1}$, which contains the predicted values of all variables from time point *t* to *t* + *T* − 1.

### 3.2 Overall architecture

**Figure 1:**
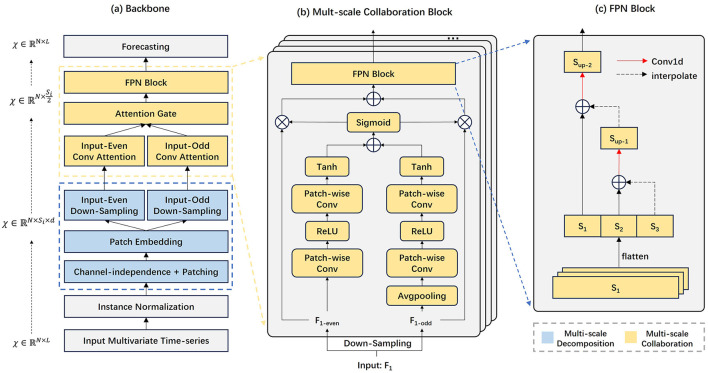
The overall architecture of AMDCnet. AMDCnet consists of a multi-scale data decomposition and collaboration block, which can capture changes in different time scales through fusion convolution blocks and learn based on up-sampling fusion representation.

The overall architecture is shown in Figure 1. The process consists of three main components: (a) a multiscale decomposition module; (b) a multiscale collaboration module based on attention gating; and (c) time series prediction. (a) AMDCnet applies a multiscale decomposition method to the input data, generating multiple time series at different resolutions. Each time scale produces two subsequences through successive down-sampling. (b) The Parallel Fusion Convolution Block processes these subsequences with distinct convolutional filters to extract both local and global features of the time series. The features at different resolutions are subsequently upsampled, balanced by attentional gating, and reaggregated into a new representation of the sequence. These paired subsequences are then reincorporated into the original time series as residuals. (c) Finally, a fully connected network is employed as a decoder to predict the time series.

### 3.3 Multi-scale decomposition

Our proposed Multi-scale Data decomposition approach transforms time series data into multi-scale data inputs. The input data *X*_*t* − *L*:*t*_ represents observations from time *t* − *L* to *t* − 1. We process this data using the normalization function RevIN (Kim et al., 2021) which has been shown to enhance the training efficiency of the model and effectively mitigate data distribution drift. The normalization process is defined as:


(1) $$\begin{array}{l} \begin{array}{c} X_{\text{in}}=\text{RevIN}\left(X_{t-L:t}\right) \end{array} \end{array}$$

**Figure 2:**
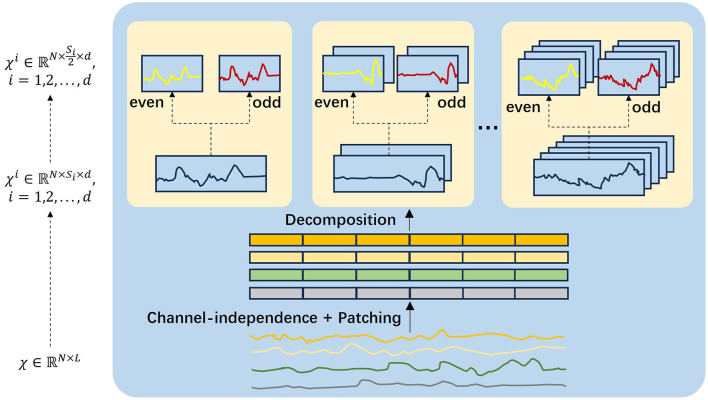
Multi-scale decomposition block. The input sequence is decomposed and converted to a parity subsequence at multiple resolutions.

The multi-scale decomposition process is shown in Figure 2. Inspired by DCdetector, we use channel independence and sub-patch methods to transform the time series into multiple time scales. For a selected set of time scales {*s*_1_, ⋯ , *s*_*k*_}, we reshape the multivariate time series inputs $X_{\text{in}}\in {\mathbb{R}}^{L\times N}$ into a 3D tensor, creating representations for different time scales using the following equations:


(2) $$\begin{array}{l} \begin{array}{c} X^{i}=\text{Reshap}{\text{e}}_{s_{i},f_{i}}\left(X_{\text{in}}\right),\;s_{i}=\frac{L}{f_{i}} \end{array} \end{array}$$

Here, $X^{i}\in {\mathbb{R}}^{N\times s_{i}\times f_{i}}$ denotes the reshaped representation for the time scale *s*_*i*_. Where *L* is the length of the sequence, and *f*_*i*_ is the scale partition factor. The *f*_*i*_ factor is then embedded into a vector of size *d*_model_, represented as *X*_emb_ and computed as follows:


(3) $$\begin{array}{l} \begin{array}{c} {\text{X}}_{\text{emb}}=\text{Conv1D}\left({\text{X}}^{i}\right)+\text{P}\text{E} \end{array} \end{array}$$

We utilize a one-dimensional convolutional filter to project *X*^*i*^ into a *d*_model_-dimensional matrix. $\text{P}\text{E}\in {\mathbb{R}}^{d_{\text{model}}\times L}$ represents the positional embedding of the input *X*^*i*^. The down-sampling decomposition process involves decomposing $X_{\text{emb}}^{i}$ into $X_{\text{odd}}^{i}\in {\mathbb{R}}^{N\times \frac{s_{i}}{2}\times d_{\text{model}}}$ and $X_{\text{even}}^{i}\in {\mathbb{R}}^{N\times \frac{s_{i}}{2}\times d_{\text{model}}}$. These decomposed sequences are then used as input matrices for the multi-scale Fusion Block.

### 3.4 Multi-scale collaboration

We propose a novel attention-gate-based multiscale collaborative block to capture both global information representations and local dependencies of sequences across different time scales. Specifically, we apply distinct information characterizations to the parity subsequences obtained from downsampling. In contrast to TCNs, which employ a single shared convolution filter at each layer, our fused convolutional module enhances feature extraction by aggregating information from subsequences decomposed at different time scales, offering both local and global perspectives of the time series at varying temporal resolutions. Unlike TCNs with shared convolutional filters, our fused convolutional module not only extracts features across multiple time scales through a diverse set of convolutional filters but also achieves a larger receptive field, akin to extended convolution.

The odd-even division mechanism is designed to enhance temporal feature structural symmetry on top of multi-scale sequences. This partitioning ensures alternating coverage of two subsequences along the temporal axis, thereby avoiding information leakage and contextual disruption. Unlike the odd sequence, the even sequence achieves global information extraction by adjusting the size of the spatial pool, thus reducing the spatial dimension of each channel of $X_{\text{odd}}^{i}$ to a one-dimensional vector with global information. The global channel context is computed as follows:


(4) $$\begin{array}{l} \text{Global}\left(X_{\text{odd}}^{i}\right)=B\left(\text{PWCon}{\text{v}}_{2}\left(\delta \left(B\left(\text{PWCon}{\text{v}}_{1}\left(Avg\left(X_{\text{odd}}^{i}\right)\right)\right)\right)\right)\right) \end{array}$$

Here, Avg(·) denotes average pooling, and patchwise convolution PWConv is used for local channel context aggregation. The kernel sizes of PWConv_1_ and PWConv_2_ are $\left(d\times C\right)\times \frac{C}{r}\times 1$, where r is the channel reduction factor, B represents the BatchNorm, and δ denotes the Rectified Linear Unit (ReLU). The local channel context branching structure is implemented by PWConv and is computed as follows:


(5) $$\begin{array}{l} \begin{array}{c} \text{Local}\left(X_{\text{even}}^{i}\right)=B\left(\text{PWCon}{\text{v}}_{2}\left(\delta \left(B\left(\text{PWCon}{\text{v}}_{1}\left(X_{\text{even}}^{i}\right)\right)\right)\right)\right) \end{array} \end{array}$$

We then summarize the global and local scale feature information through the attention-gate unit, using the following equation:


(6) $$\begin{array}{l} \begin{array}{c} w=\sigma \left(\text{Local}\left(X_{\text{even}}^{i}\right)⊕\text{Global}\left(X_{\text{odd}}^{i}\right)\right), \end{array} \end{array}$$

(7) $$\begin{array}{l} X_{\text{out}}^{\hat{i}}=w⊗\text{Global}\left(X_{\text{odd}}^{i}\right)+\left(1-w\right)⊗\text{Local}\left(X_{\text{even}}^{i}\right) \end{array}$$

Here, ⊕ denotes the broadcast addition which generates an attentional representation incorporating both local and global context. The function σ is a Sigmoid function that serves as a gating unit to regulate the weights of local and global representations. ⊗ denotes the dot product. Finally $X_{\text{out}}^{\hat{i}}\in {\mathbb{R}}^{N\times \frac{s_{i}}{2}}$ is used as a fused feature. To advance our model, we need to integrate tensors of different scales $X_{\text{out}}^{\hat{1}},X_{\text{out}}^{\hat{2}}\cdots \;,X_{\text{out}}^{\hat{k}}$. The Feature Pyramid Networks (FPN) structure, renowned for its ability to capture features at multiple scales, is widely used in target detection and semantic segmentation due to its powerful feature extraction capabilities. Inspired by the FPN, we employ a pyramid structure to aggregate different time scales, enabling our model to integrate and leverage information from various temporal resolutions effectively.


(8) $$\begin{array}{l} {\hat{\text{X}}}_{\text{out}}=\text{Interp}(\ldots (\text{Interp}(X_{\text{out}}^{\hat{1}})+X_{\text{out}}^{\hat{2}})+\ldots )+X_{\text{out}}^{\hat{k}} \end{array}$$

In this process, Interp(·) is an interpolation operation where we recover high-resolution features step-by-step by up-sampling through linear interpolation. This method fuses multiple resolution feature layers, effectively capturing the multi-scale dynamic information of the data. This blending strategy promotes the integration of multi-scale features into the subsequent layers, enhancing the model's ability to utilize diverse temporal information.

### 3.5 Time series forecasting

The model utilizes a linear projection to map ${\hat{\text{X}}}_{\text{out}}\in {\mathbb{R}}^{N\times L}$ to the ${\hat{\text{X}}}_{t:t+T}\in {\mathbb{R}}^{N\times T}$ for prediction. The projection process is described as follows:


(9) $$\begin{array}{l} \begin{array}{c} {\hat{\text{X}}}_{t:t+T}={\hat{\text{X}}}_{\text{out}}{\text{W}}_{\text{t}}+\text{b} \end{array} \end{array}$$

Here, ${\text{W}}_{\text{t}}\in {\mathbb{R}}^{L\times T}$ and **b** ∈ ℝ^*T*^ are learnable parameters. ${\hat{\text{X}}}_{t:t+T}$ is the final prediction.

## 4 Experiments

### 4.1 Datasets

We validate the performance of AMDCnet on eight widely used and recognized benchmark datasets, including Weather, Traffic, Electricity, ETT (ETTh1, ETTh2, ETTm1, ETTm2), and Exchange. Table 1 provides a summary of the statistics for these datasets and the detailed information of each dataset is as follows:

- *Weather* (Angryk et al., 2020) consists of time series data for 21 meteorological metrics, collected every 10 min by the Max Planck Biogeochemical Research Weather Station in 2020.
- *Traffic* (Chen et al., 2001) records hourly road occupancy rates measured by 862 sensors on San Francisco Bay Area freeways, spanning January 2015 to December 2016.
- *Electricity* (Khan et al., 2020) contains hourly electricity consumption data for customers from 2012 to 2014.
- *ETT* (Zhou et al., 2021) comprises electric transformer data from July 2016 to July 2018, including load and oil temperatures.
- *Exchange* (Lai et al., 2018) tracks daily exchange rates from 1990 to 2016 for eight countries.

**Table 1:** Statistics of popular datasets for the benchmark.

**Dataset**	**Weather**	**Traffic**	**Electricity**	**ETTh1/2**	**ETTm1/2**	**Exchange**
Variates	21	862	321	7	7	8
Timesteps	52,696	17,544	26,304	17,420	69,680	7,588
Granularity	10 min	1 h	1 h	1 h	15 min	1 day

### 4.2 Baselines

To validate the effectiveness of the AMDCnet model, we compared it with six well-known time-series forecasting models, including the latest multi-scale model MSGnet (Cai et al., 2024), dominant Transformer-based models: TimesNet (Wu et al., 2022), FEDformer (Zhou et al., 2022), Non-stationary Transformer (Liu et al., 2022), and Informer (Zhou et al., 2021). Additionally, we also included a non-Transformer-based model, DLinear (Zeng et al., 2023).

### 4.3 Experimental setups

For a fair comparison, the same experimental setup was used for all models. We used the ADAM (Kingma, 2014) optimizer to train the models, with the learning rate set to 1e-4, training epochs set to 10, and mean square error (MSE) as the training loss function. The baseline used relevant data from the paper TimesNet or the official code. Our model is based on PyTorch (Paszke et al., 2019), and all experiments were conducted using NVIDIA GeForce RTX 3090 24GB GPUs.

### 4.4 Experimental results

Table 2 presents the main experimental results of all models across eight datasets, with the best and second-best results for each scenario (dataset, level, and metric) highlighted in bold and underlined. AMDCnet demonstrates outstanding performance in long-term time-series forecasting. Specifically, AMDCnet outperforms existing benchmark methods, achieving 44 best results and 10 second-best results out of 64 cases. Compared to the state-of-the-art multi-scale model MSGNet, AMDCnet shows a near-complete improvement in performance across most datasets (Weather, Traffic, Electricity, ETTh1, ETTh2, ETTm1), with moderate or approximately equivalent improvements observed on a few datasets (ETTm2, Exchange). In terms of the average Mean Squared Error (MSE) across all datasets, AMDCnet significantly reduces the MSE by 6.4% (from 0.357 to 0.334) compared to MSGNet, by 7.2% (from 0.360 to 0.334) compared to the multi-scale Transformer-based model TimesNet, and by 12.7% (from 0.383 to 0.334) compared to DLinear. Overall, AMDCnet surpasses the current state-of-the-art multi-scale model MSGNet and other models.

**Table 2:** Comparative results of long-term forecasting performance on eight real datasets.

**Models**		**Ours**		**MSGNet**		**TimesNet**		**DLinear**		**FEDformer**		**Non-stationary**		**Informer**	
**Metrics**		**MSE**	**MAE**	**MSE**	**MAE**	**MSE**	**MAE**	**MSE**	**MAE**	**MSE**	**MAE**	**MSE**	**MAE**	**MSE**	**MAE**
Weather	96	**0.155**	**0.206**	0.163	0.212	0.172	0.220	0.196	0.255	0.238	0.314	0.173	0.223	0.354	0.405
	192	**0.207**	**0.253**	0.212	0.254	0.219	0.261	0.237	0.296	0.275	0.329	0.245	0.285	0.419	0.434
	336	**0.267**	**0.297**	0.272	0.299	0.280	0.306	0.283	0.335	0.339	0.377	0.321	0.338	0.583	0.543
	720	0.347	**0.347**	0.350	0.348	0.365	0.359	**0.345**	0.381	0.389	0.409	0.414	0.410	0.916	0.705
Traffic	96	**0.447**	**0.296**	0.589	0.343	0.593	0.321	0.650	0.396	0.576	0.359	0.612	0.338	0.733	0.410
	192	**0.465**	**0.307**	0.616	0.363	0.617	0.336	0.598	0.370	0.610	0.380	0.613	0.340	0.777	0.435
	336	**0.478**	**0.314**	0.642	0.372	0.629	0.336	0.605	0.373	0.608	0.375	0.618	0.328	0.776	0.434
	720	**0.517**	**0.335**	0.689	0.403	0.640	0.350	0.645	0.394	0.621	0.375	0.653	0.355	0.827	0.466
Electricity	96	**0.154**	**0.256**	0.165	0.274	0.168	0.272	0.193	0.308	0.186	0.302	0.169	0.273	0.304	0.393
	192	**0.169**	**0.266**	0.184	0.292	0.184	0.289	0.201	0.285	0.197	0.311	0.182	0.286	0.327	0.417
	336	**0.188**	**0.286**	0.195	0.302	0.198	0.300	0.209	0.301	0.213	0.328	0.200	0.304	0.333	0.422
	720	**0.214**	**0.310**	0.231	0.332	0.220	0.320	0.245	0.333	0.233	0.344	0.222	0.321	0.351	0.427
ETTh1	96	0.388	**0.396**	0.390	0.411	**0.380**	0.402	0.386	0.400	0.376	0.419	0.513	0.491	0.941	0.769
	192	0.438	**0.425**	0.442	0.442	0.436	0.429	0.437	0.432	**0.420**	0.448	0.534	0.504	1.007	0.786
	336	**0.479**	**0.445**	0.480	0.468	0.491	0.469	0.481	0.459	0.459	0.465	0.588	0.535	1.038	0.784
	720	**0.484**	**0.470**	0.494	0.488	0.521	0.500	0.519	0.516	0.506	0.507	0.643	0.616	1.144	0.857
ETTh2	96	**0.310**	**0.355**	0.328	0.371	0.340	0.374	0.333	0.387	0.358	0.397	0.476	0.458	1.549	0.952
	192	**0.388**	**0.403**	0.402	0.414	0.402	0.419	0.477	0.476	0.429	0.439	0.512	0.493	3.792	1.542
	336	**0.429**	**0.438**	0.435	0.443	0.452	0.452	0.594	0.541	0.496	0.487	0.552	0.551	4.215	1.642
	720	0.458	0.461	**0.417**	**0.441**	0.462	0.468	0.657	0.450	0.463	0.474	0.562	0.560	3.656	1.619
ETTm1	96	0.337	0.371	**0.319**	**0.366**	0.340	0.377	0.345	0.372	0.379	0.419	0.386	0.398	0.626	0.560
	192	**0.355**	0.393	0.376	0.397	0.374	**0.387**	0.380	0.389	0.426	0.441	0.459	0.444	0.725	0.619
	336	**0.409**	**0.409**	0.417	0.422	0.410	0.413	0.413	0.413	0.445	0.459	0.495	0.464	1.005	0.740
	720	**0.474**	**0.444**	0.481	0.478	0.478	0.450	0.474	0.453	0.543	0.490	0.585	0.516	1.133	0.845
ETTm2	96	**0.174**	**0.259**	0.177	0.262	0.183	0.271	0.193	0.293	0.203	0.287	0.192	0.274	0.355	0.462
	192	0.256	0.315	**0.247**	**0.307**	0.248	0.309	0.284	0.362	0.269	0.328	0.280	0.339	0.595	0.586
	336	0.324	0.356	0.312	**0.346**	**0.304**	0.348	0.369	0.427	0.325	0.366	0.334	0.361	1.270	0.871
	720	0.413	0.406	0.414	0.403	**0.385**	**0.400**	0.554	0.522	0.421	0.415	0.417	0.413	3.001	1.267
Exchange	96	0.092	**0.215**	0.102	0.230	0.107	0.234	**0.088**	0.218	0.148	0.278	0.111	0.237	1.847	0.752
	192	0.189	**0.311**	0.195	0.317	0.226	0.344	**0.176**	0.315	0.271	0.380	0.219	0.335	1.204	0.895
	336	0.354	0.431	0.359	0.436	0.347	0.448	**0.313**	**0.427**	0.46	0.500	0.421	0.476	1.672	1.036
	720	0.864	0.696	0.94	0.738	0.964	0.746	**0.839**	**0.695**	1.195	0.841	1.092	0.769	2.478	1.310
#Rank-1st		**44**		7		5		7		1		0		0	

The past sequence length of each dataset *L* = 96 and the prediction length *T* ∈ {96,192,336,720}. Bold and underlined are labeled as the best and second-best results.

**Figure 3:**
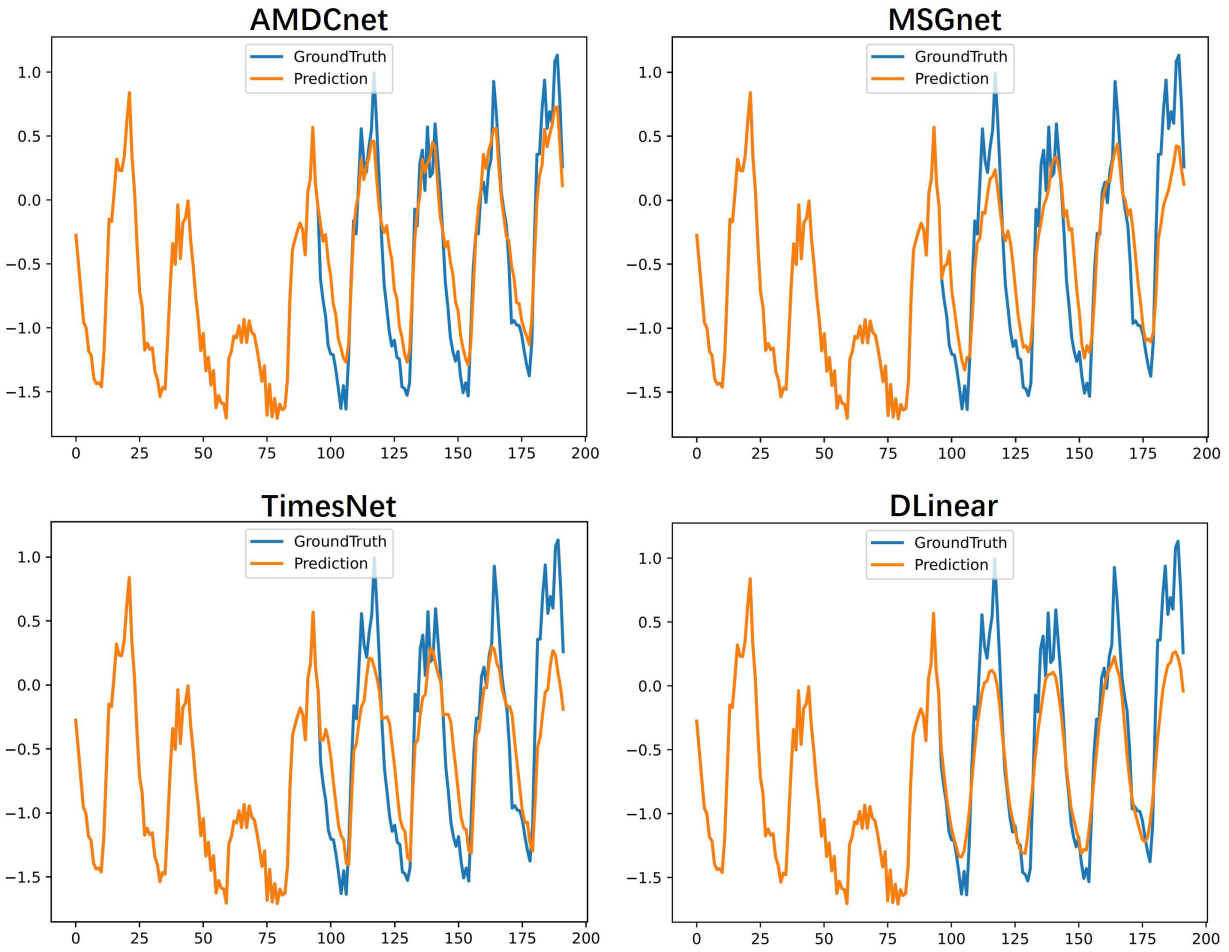
Visualization of the predicted results for the Electricity dataset: the blue line shows the true value and the orange line shows the predicted result.

We visualize the forecasting performance of AMDCnet, MSGNet, TimesNet, and DLinear on the Electricity dataset to evaluate their long-term prediction capabilities. As shown in Figure 3, AMDCnet exhibits high prediction accuracy and effectively captures the variation patterns in high-frequency fluctuation regions, with its predicted curve closely aligning with the true values. MSGNet and TimesNet generally perform well, but face difficulties in accurately predicting extreme values, while DLinear performs poorly, showing low prediction accuracy even in non-extreme regions. Although MSGNet, TimesNet, and DLinear are capable of learning the data change patterns in high-frequency fluctuation areas, demonstrating their ability to model the seasonal and trend characteristics of the data, they struggle to accurately predict peaks, leading to substantial deviations from the true values. In contrast, AMDCnet shows high prediction accuracy, highlighting its ability to handle complex and dynamic time-series data.

A comprehensive analysis of the adaptive weighting mechanism revealed that the learned weights predominantly fluctuate between 0.3 and 0.7, with a mean value of ~0.49. This oscillatory behavior suggests that the model effectively captures distinct weighting patterns from odd and even sequences. To assess the impact of adaptive weighting, we compared it against a fixed-weight baseline (set to 0.5, equivalent to a uniform distribution across odd and even sequences). The results demonstrated a reduction in MSE of 0.03 on the ECL dataset (input length: 96, prediction horizon: 96). While the improvement is modest, it underscores the efficacy of the attention-based gating mechanism.

## 5 Model analysis and ablation study

### 5.1 Analysis: past sequence length

AMDCnet achieved state-of-the-art performance in experiments with *L* = 96. To further evaluate its effectiveness, we extended the assessment to *L* = 336. The Electricity and Weather datasets, where AMDCnet demonstrated superior results in the main experiment, were selected for this evaluation. The prediction lengths remain consistent with those used at *L* = 96, i.e., *T* ∈ {96, 192, 336, 720}, and are compared against the current state-of-the-art models, MSGNet and TimesNet.

**Figure 4:**
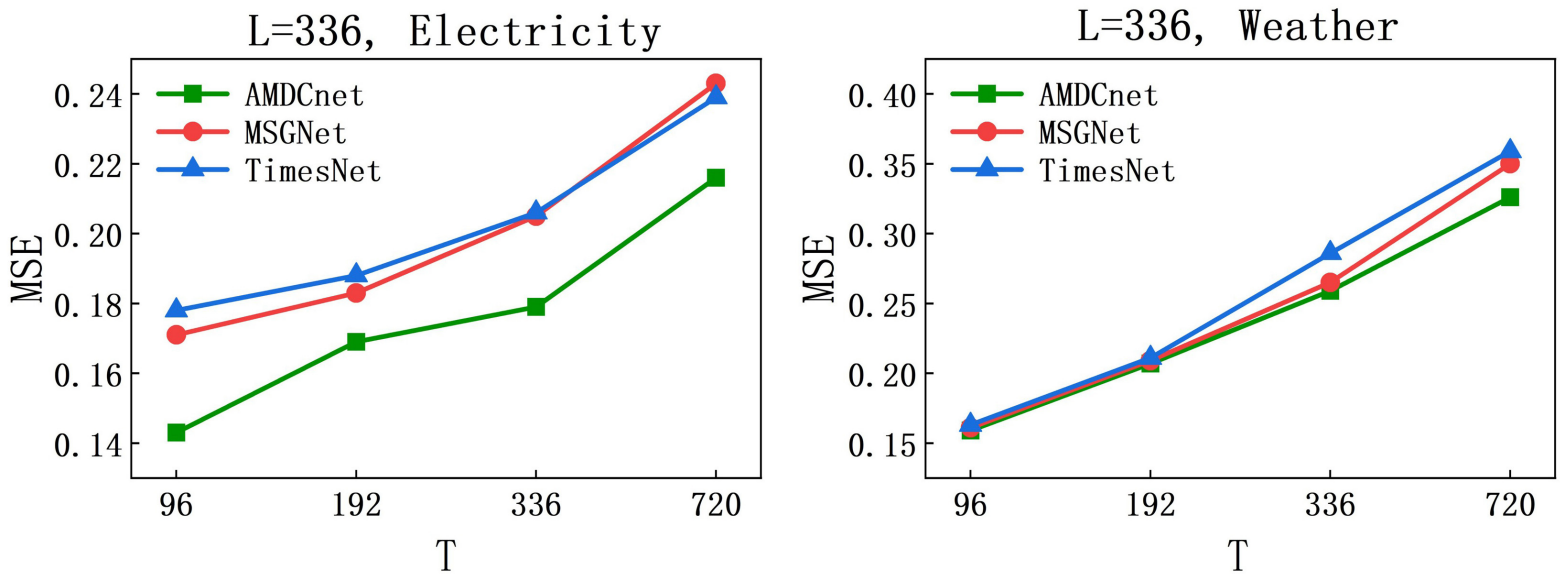
MSE at different prediction lengths on Electricity and Weather datasets, *L* = 336 and *T* ∈ {96, 192, 336, 720}.

The obtained MSE results are visualized in Figure 4. The curves indicate that, regardless of the methods and datasets used, the MSEs consistently increase as the prediction sequence lengthens. This trend is consistent with the performance observed at *L* = 96, where the models' predictive accuracy gradually declines with increasing prediction length. In terms of model comparison, AMDCnet outperforms both MSGNet and TimesNet in the Electricity dataset and slightly surpasses MSGNet and TimesNet in the Weather dataset at *L* = 336. Collectively, AMDCnet maintains a level of excellence beyond the current SOTA MSGNet.

### 5.2 Analysis: training dataset

**Figure 5:**
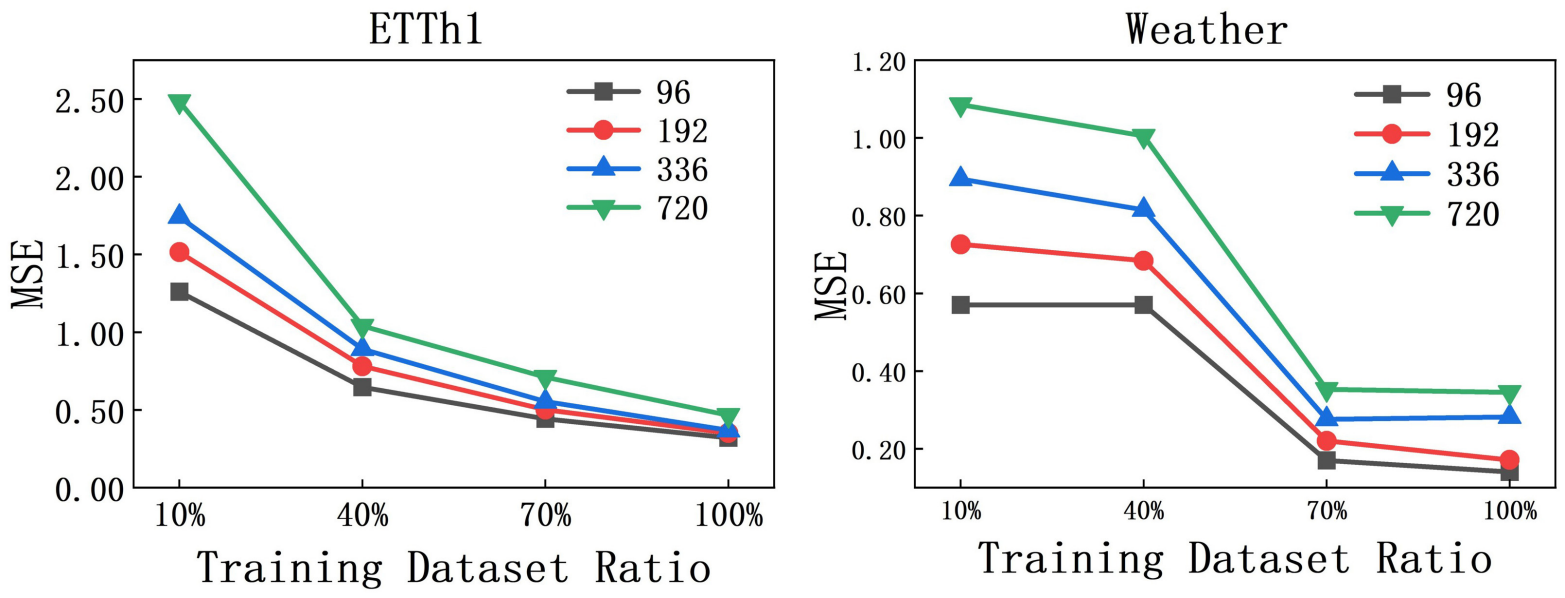
MSE on ETTh1 and Weather with different Training dataset ratio.

The experiment on the impact of the size of the training set on the performance of the model is mainly to evaluate whether the model itself is effective, and at the same time help us observe whether a full dataset is needed to train the model. We investigated the effect of training set size on AMDCnet's performance using the ETTh1 and Weather datasets. The parameter settings were consistent with previous experiments, with *L* = 96 and *T* ∈ {96, 192, 336, 720}. As shown in Figure 5, for the ETTh1 dataset, the MSE is significantly higher when the training set size is only 10%. However, as the training set proportion increases, the MSE steadily decreases and eventually stabilizes. A similar trend is observed for the Weather dataset, where the MSE also decreases as the training set size increases. However, unlike ETTh1, where the MSE quickly stabilizes, the MSE for the Weather dataset drops rapidly between 40 and 70% of the training set size, with little change beyond this point. Empirical results show that comparable model performance can be achieved without using the complete training dataset, especially in specific data fields such as meteorological observation. This observation result indicates that strategically reducing the cardinality of the training set can achieve the effect of reducing the training time while maintaining the validity of the model.

### 5.3 Analysis: computational efficiency

To evaluate training efficiency, we performed experiments on the electricity dataset, comparing AMDCnet with Transformer-based models (MSGnet, TimesNet, FEDformer) and DLinear. All models were trained under identical hyperparameters, including a historical sequence length of 96, a prediction horizon of 96, and a batch size of 64. As shown in Table 3, AMDCnet exhibits faster training speeds than all Transformer-based counterparts. The latter typically demand longer training durations due to the computational overhead of their self-attention mechanisms. In contrast, AMDCnet leverages a convolutional neural network architecture, which confers a substantial efficiency advantage in training time. DLinear, a non-Transformer baseline included in this study, achieved an average training time of merely 5.63 seconds per epoch, surpassing all other models in efficiency. However, this result stems primarily from its simplistic linear structure, which lacks the representational complexity of convolutional or Transformer-based approaches. Overall, AMDCnet attains the second-highest training efficiency while maintaining superior experimental performance.

**Table 3:** Average training time of the models.

**Model**	**AMDCnet**	**MSGnet**	**TimesNet**	**DLinear**	**FEDformer**
AVGTimes/epoch(s)	67.46	107.65	186.84	5.63	79.43

### 5.4 Ablation: fused convolution

The Fused Convolution module integrates local and global information representations along with an attention-gating unit. For comparison purposes, we replaced this module with a shared convolution kernel, which does not require the moderation of the gating unit and does not differentiate between local and global information: *w*/*o* *FB*. As shown in Table 4, the performance degrades after this replacement for the Weather, Traffic, and ETTh1 datasets. However, there is a slight improvement in the performance for the ETTm2 dataset at prediction lengths of 336 and 720. This could be attributed to overfitting during the fusion convolution process, which leads to an imbalanced allocation of gate unit weights. Overall, the decline in performance validates the effectiveness of the fusion convolution module in multi-scale collaborative work.

**Table 4:** Ablation analysis of weather, traffic, ETTh1, and ETTh2 datasets.

**Models**		**AMDCnet(base)**		**w/o FB**		**w/o FC**	
**Metrics**		**MSE**	**MAE**	**MSE**	**MAE**	**MSE**	**MAE**
Weather	96	**0.155**	**0.206**	0.162	0.215	0.163	0.214
	192	**0.207**	**0.253**	0.212	0.254	0.211	0.256
	336	**0.267**	**0.297**	0.269	0.298	0.267	0.296
	720	**0.347**	**0.347**	0.348	0.347	0.347	0.348
Traffic	96	**0.447**	**0.296**	0.522	0.358	0.505	0.337
	192	**0.465**	**0.307**	0.519	0.351	0.504	0.338
	336	**0.478**	**0.314**	0.526	0.352	0.517	0.341
	720	**0.517**	**0.335**	0.568	0.373	0.556	0.361
ETTh1	96	**0.388**	**0.396**	0.396	0.403	0.392	0.399
	192	**0.438**	**0.425**	0.445	0.431	0.44	0.426
	336	**0.479**	**0.445**	0.483	0.45	0.481	0.447
	720	**0.484**	**0.470**	0.499	0.479	0.484	0.471
ETTm1	96	**0.337**	**0.371**	0.338	0.371	0.34	0.377
	192	**0.355**	0.393	0.375	**0.387**	0.381	0.4
	336	0.409	0.409	**0.405**	**0.407**	0.417	0.419
	720	0.474	0.444	**0.470**	**0.443**	0.496	0.462

Results represent the prediction length {96, 192, 336, 720}, with the best performance highlighted in bold black.

### 5.5 Ablation: FPN block

Feature Pyramid Network (FPN) is a crucial method for multi-scale feature fusion, combining deep and shallow features through a feature pyramid structure and lateral connections. This allows the network to capture target information at varying scales in the image more comprehensively. In AMDCnet, we utilized interpolated upsampling for FPN fusion. During the ablation experiments, we designed a fully connected module to directly fuse the data as a comparison to verify the FPN block's capability in capturing multi-scale features: *w*/*o* *FC*. As shown in Table 4, the model's performance consistently degrades across almost all datasets after replacing the FPN block. This indicates that the FPN effectively fuses multi-scale features under different datasets, validating its ability to capture multi-scale features—consistent with mainstream experimental findings. Additionally, it demonstrates that the upsampling interpolation fusion method, employed as part of AMDCnet's multi-scale collaboration module, plays a critical role in the post-decomposition synergy.

## 6 Conclusion

In this paper, we propose a novel network for long-term time series prediction called Attention-gate-based Multiscale Decomposition and Collaboration Network, built upon multiscale time series processing techniques. AMDCnet leverages multiscale decomposition of time series and collaboration through gating units for feature fusion. Specifically, we incorporate channel independence and sub-patch strategies in the multiscale decomposition process, where the sequence is downsampled and processed by a multiscale fusion convolution module to learn both local and global sequence features. After weight assignment via the gating unit, the data enters the FPN module for feature fusion. Extensive experiments on eight real-world datasets demonstrate that AMDCnet outperforms existing models and effectively captures dependencies across different time scales. Additionally, ablation studies on the fusion convolution module and the FPN module further confirm the effectiveness of AMDCnet. In future work, we aim to explore additional methods further to improve the accuracy of AMDCnet in time series prediction.

## Data Availability

Publicly available datasets were analyzed in this study. This data can be found at: https://drive.google.com/drive/folders/1ZOYpTUa82_jCcxIdTmyr0LXQfvaM9vIy.
